# Supplementary material for: A Narrative Review of the Strengths and Limitations of Real-World Evidence in Comparison to Randomized Clinical Trials: What Are the Opportunities in Thoracic Oncology for Real-World Evidence to Shine?
Source: Curr Oncol. 2025 Nov 10;32(11):629. doi: 10.3390/curroncol32110629 (PMC12651554; doi:10.3390/curroncol32110629)
Supplement: Supplementary file 1 [file curroncol-32-00629-s001.zip › curroncol-3866134-supplementary.pdf]

Supplementary Figure S1. Literature search strategy

1. exp Randomized Controlled Trials as Topic/
2. rct.ti,ab.
3. randomized control* trial.mp.
4. 1 or 2 or 3
5. "real world design".mp.
6. "real world evidence".mp.
7. rwe.ti,ab.
8. real-world evidence.mp.
9. real world design.mp.
10. 5 or 6 or 7 or 8 or 9
11. 4 and 10
12. exp bias/
13. Publication Bias/
14. strength*.mp.
15. limitation*.mp.
16. (pros or cons).mp. [mp=title, abstract, original title, name of substance word, subject heading word, floating sub-heading word, keyword heading word, organism supplementary concept word, protocol supplementary concept word, rare disease supplementary concept word, unique identifier]
17. 12 or 13 or 14 or 15 or 16
18. 11 and 17
19. Research Design/
20. 11 or 19
21. 17 and 20
22. 4 or 19
23. 10 and 17 and 22
24. limit 23 to meta analysis
